# A New Species of the Acanthocephalan Genus *Filisoma* (Cavisomidae) from Perciform Fishes in Rio de Janeiro, Brasil

**DOI:** 10.2478/s11686-018-00019-3

**Published:** 2019-02-11

**Authors:** Viviane S. Costa Fernandes, Omar Amin, Juliana N. Borges, Cláudia P. Santos

**Affiliations:** 1 Laboratório de Avaliação e Promoção da Saúde Ambiental (LAPSA)-Fundação Oswaldo Cruz, Av. Brasil 4365, Rio de Janeiro, 21040-360 Brazil; 2 Institute of Parasitic Diseases, Scottsdale, AZ USA

**Keywords:** Acanthocephala, *Filisoma caudata* n. sp., Fish, *Kyphosus*, Systematic, Taxonomy

## Abstract

**Background:**

Twelve species of *Filisoma* Van Cleave, 1928 are recognized parasitizing tropical and subtropical fish. Four of these species were described from kyphosid fish and it has been suggested that a co-speciation may have occurred among species of *Kyphosus* Lacepède, 1801 and *Filisoma*, which could provide valuable information about the evolution history of this host–parasite system.

**Purpose:**

During a survey of the helminth fauna of *Kyphosus sectatrix* (Linnaeus, 1758) and *Kyphosus incisor* (Cuvier, 1831) (Kyphosidae Jordan, 1887) off Rio de Janeiro coast, a new species of *Filisoma* was found and is described herein based on morphological, genetic, and ultrastructural data.

**Methods:**

Fish were obtained off Rio de Janeiro coast, Brazil. The parasites found in the intestine were measured and drawings were made with a drawing tube. Type specimens were deposited at the Helminthological Collection of Oswaldo Cruz Institute (CHIOC). The ultrastructure was studied using scanning electron microscope. The genetic analysis included the study of the partial sequences of 18S, ITS1, 5.8S and 28S rDNA, and the mitochondrial cytochrome c oxidase 1 gene (*cox* 1), with phylogenetic reconstructions based on the maximum likelihood analysis.

**Results:**

*Filisoma caudata* n. sp. is characterized by a proboscis with 16‒18 longitudinal rows of 38‒45 hooks each. Hooks are uniform in shape dorsoventrally, gradually decreasing in size towards the base of the proboscis. Anterior hooks are 30‒45 μ long, middle hooks 30‒35 μ long and 5 basal transversal hooks 20‒30 μ long. The new species is differentiated from the closest species *Filisoma filiformis* Weaver and Smales, 2013 by the size and distribution of hooks, apart from having a subterminal vulva and a curved posterior trunk end (tail) measuring 500‒1,000 long. Phylogenetic analysis based on 18S, 28S rDNA and mtDNA-cox1 markers grouped the new species with *Filisoma bucerium* Van Cleave, 1940 and *Filisoma rizalinum* Tubangui and Masiluñgan, 1946 showing a close relationship between these species of Cavisomidae Meyer, 1932 and Echinorhynchidae Cobbold, 1879; the latter represented by species of *Acanthocephalus Koelreuther*, 1771. The new species can be differentiated from others on morphological and molecular basis. A key to the 13 species of *Filisoma* Van Cleave, 1928 is provided.

**Conclusion:**

*Filisoma caudata* n. sp. is described herein based on morphological, genetic, and ultrastructural data. The topologies of obtained phylogenies suggest that species of Echinorhynchidae should be reevaluated since the family is considered paraphyletic in all analyses conducted.

## Introduction

Acanthocephalans of the genus *Filisoma* Van Cleave, 1928 have been described from fish from tropical and subtropical waters, including species of the family Kyphosidae Jordan, 1887 [1, 2]. Twelve species are recognized within the genus [3] including the type species *Filisoma bucerium* Van Cleave, 1940; *F. acanthocybii* Wang, Wang & Wu, 1993; *F. atropi* Wang, 1988; *Filisoma fidum* Van Cleave & Manter 1947; *Filisoma filiformis* Weaver and Smales 2013; *F. indicum* Van Cleave, 1928; *F. inglisi* Gupta & Naqvi, 1984; *F. longcementglandatus* Amin & Nahhas, 1994; *F. micracanthi* Harada, 1938; *F. oplegnathi* Wang, 1988; *Filisoma rizalinum* Tubangui & Masiluñgan, 1946 and *F. scatophagusi* Datta & Soota, 1962 (see especially [2, 4, 5]).

Four of these species were described from kyphosid fish and it has been suggested that a co-speciation may have occurred among species of *Kyphosus* Lacepède, 1801 and *Filisoma* which could provide valuable information about the evolution history of this host–parasite system [1, 6, 7]. Although studies that use the integrative taxonomy (using different tools that includes morphological, ultrastructural, biochemical, molecular, and behavioral studies to delimit and characterize species) have been increasing in the last few years [8], the number of studies using this approach is still scarce [9, 10]).

During a survey of the helminth fauna of *Kyphosus sectatrix* (Linnaeus, 1758) and *Kyphosus incisor* (Cuvier, 1831) off Rio de Janeiro coast, a new species of *Filisoma* was found and is described herein based on morphological, genetic, and ultrastructural data.

## Materials and Methods

### Ethical Statement

Collections in this study were authorized by the Brazilian Institute of Environment and Renewable Natural Resources (IBAMA, license no. 15898-1).

### Fish Collection

A total of 22 specimens of *K. incisor* were examined from October 2013 to March 2015; nine were acquired from fishermen of Copacabana beach (22°59′08″S, 43°11′18″W) and 13 were obtained from local fish markets. The mean total length of fish was 46 ± 5 (35–53) cm and mean weight was 1672 ± 549 (730–2370) g. Acanthocephalans from a single specimen of *K. sectatrix* measuring 30 cm long and weighing 525 g, collected in 2005 at Ilha Grande Bay (23°04′04.62″S, 44º13′31.95″W) were also examined for comparison. Fish were identified according to Froese and Pauly [11].

### Light Microscopy

The parasites found in the intestine were washed in physiological saline (0.7%) and fixed in AFA, 4% formalin or 70% alcohol. Some acanthocephalans were stained with alcohol chloride carmine, cleared in clove oil and mounted in Canada balsam. Observations were based on the specimens collected from two hosts: *K. incisor* and *K*. *sectatrix* and measurements are given in micrometres, with range in parentheses, unless otherwise stated; holotype measurements are in brackets. Drawings were made with a drawing tube and redrawn using Adobe Illustrator CS6 [12]. The prevalence, intensity, mean intensity and mean abundance follow Bush et al. [13]. Specimens were deposited at the Helminthological Collection of Oswaldo Cruz Institute (CHIOC).

### Scanning Electron Microscopy (SEM)

Specimens fixed in AFA or 4% formalin were washed in 0.1 M Na-cacodylate buffer, post-fixed for 40 min in a solution of 1% osmium tetroxide in 0.8% potassium ferrocyanide in 0.1 M Na-cacodylate buffer, dehydrated in an ascending alcohol series, dried by the critical point method with CO_2_, and sputter-coated with gold 60 nm. Samples were examined using a JEOL JSM 6390 LV scanning electron microscope (JEOL Ltd., Tokyo, Japan) at the Electron Microscopy Platform, Fundação Oswaldo Cruz.

### Genetic Analysis

DNA was extracted using the phenol–chloroform method as described by Billings et al. [14] and a set of primers were used to amplify different regions of the DNA. The partial 28S rDNA gene was amplified by PCR using the primers C1 (5′-ACC CGC TGA ATT TAA GCA T-3′) and D2 (5′-TGG TCC GTG TTT CAA GAC-3′) (Hassouna et al. [15], after Chisholm et al. [16]). For partial 18S, ITS1 and 5.8S, the primers S1 (5′-TTC CGA TAA CGA ACG AGA CT-3′) and H7 (5′-GCT GCG TTC TTC ATC GAT ACT CG-3′) [17] were used. For partial fragment of the mitochondrial cytochrome *c* oxidase 1 gene (*cox* 1) primers LCO (5′-GGT CAA CAA ATC ATA AAG ATA TTG G-3′) and HCO (5′-TAA ACT TCA GGG TGA CCA AAA AAT CA-3′) [18] were used. PCR was carried out using cycling parameters as previously described by those authors. The PCR products were analyzed by electrophoresis in 1.5% agarose gels, stained with SyberGreen (Invitrogen, Eugene, Oregon, USA) and photographed under UV transillumination. Amplified PCR products were purified with ExoSap-IT PCR Product Cleanup (USB^®^ Products Affymetrix Inc., Cleveland, Ohio, USA). DNA cycle sequencing reactions were performed using BigDye Terminator v.3.1 (Applied Biosystems, Foster City, CA, USA) and automated sequencing was done using the Sequencing Platform at the Fundacão Oswaldo Cruz-PDTIS/FIOCRUZ in Brasil. Sequences of both strands were edited and aligned using the MEGA version 7.0 software [19]. Sequences were compared to others available in the GenBank database using the BLASTN program from the National Center for Biotechnology Information (NCBI) server (http://www.ncbi.nlm.nih.gov/BLAST) [20]. The nucleotide sequences were aligned using the CLUSTAL W algorithm [21] of the MEGA 7.0 package. Maximum likelihood (ML) phylogenetic trees were inferred using the best-fit model of MEGA 7.0: the Kimura two parameters (K2P) with invariant site (I) for the 18S rDNA and for 28S rDNA and mtDNA *cox*-*1* the Hasegawa–Kishino–Yano (HKY) model including estimates of invariant sites (I) and gamma distribution. The tree was resampled by 1000 bootstrap replicates to evaluate the reliability of the groups. The sequences of *Filisoma caudata* n. sp were deposited in the GenBank as mtDNA *cox*-1 region accession numbers MH004408 and MH004410 (666 bp each); MH004409 and MH004411 (665 bp each); MH004407 (657 bp) and MH021180 (635 bp). The sequences for partial 18S rDNA included numbers MH004443 (723 bp), MH004443 (647 bp), MH004444 (651 bp) and MH004445 (453 bp). For partial 28S rDNA the accession numbers were MH004455 (670 bp) and MH004456 (513 bp). The sequences used for the phylogenetic analysis are listed in Table 1.

**Table 1:** GenBank sequences used in the phylogenetic analysis

Species	Accession numbers		
	18S rDNA	28S rDNA	mtDNA-*cox1*
*Filisoma caudata* n.sp.	MH004442-MH004445	MH004455-MH004456	MH004407-MH004411/MH021180
*Filisoma bucerium*	AF064814	AY829110	DQ089722
*Filisoma rizalinum*	JX014229	–	–
*Acanthocephalus dirus*	AY830151	–	DQ089718
*Acanthocephalus lucii*	AY830152	KM656148	AM039837/KP261016
*Acanthocephalus nanus*	–	LC100043	–
*Acanthocephalus anguillae*	–	–	AM039864
*Acanthocephalus* sp.	DQ147605	–	–
*Pseudoacanthocephalus toshimai*	LC129278	–	–
*Pseudoacanthocephalus lucidus*	LC129279	LC100041	–
*Pseudoacanthocephalus nguyenthileae*	–	KC491890	–
*Pseudoacanthocephalus bufonis*	–	KC491878	–
*Acanthocephaloides propinquus*	AY830149	–	–
*Echinorhynchus gadi*	EF107643/EF107646	–	KP261022
*Echinorhynchus truttae*	AY830156	KM656147	–
*Echinorhynchus brayi*	–	KM656151	KP261015
*Rhadinorhynchus lintoni*	JX014224	–	–
*Rhadinorhynchus pristis*	JX014226	–	JQ061132
*Pomphorhynchus tereticollis*	AY423347	–	–
*Pomphorhynchus laevis*	JX014223	–	–
*Macracanthorhynchus ingens*	AF001844	–	–
*Polyacanthorhynchus caballeroi*	–	DQ089738	DQ089724

## Results

Acanthocephala

**Palaeacanthocephala Meyer, 1931**

**Echinorhynchida Southwell & Macfie, 1925**

**Cavisomidae Meyer, 1932**

***Filisoma*** **Van Cleave, 1928**

***Filisoma caudata*** **n. sp.**

Type host: *K. incisor* (Cuvier)

Other host: *K. sectatrix* (Linn.)

Site of infection: Intestine

Type locality: Copacabana Beach, Rio de Janeiro, Brasil (22°59′08″S, 43°11′18″W)

Other locality: Ilha Grande Bay (*K. sectatrix*), Rio de Janeiro, Brasil (23°04′04.62″S, 44º13′31.95″W)

Specimens deposited: From *K. incisor* no. 39072 a–f (a-holotype, b-alotype, c–f paratypes), 39073 and 39075 a–b (vouchers). From *K. sectatrix* no. 39074 a–b (males) and c–d (females).

Etymology: The new species is named as an adjective referring to the specific name of the host.

Nine of 22 examined specimens of *K. incisor* (41%) were infected with 212 acanthocephalans in Copacabana Beach, Rio de Janeiro. Intensity was 4–87 with a mean intensity of 25 ± 28 and mean abundance: 10 ± 21. One specimen of *K. sectatrix* was also found heavily infected. The acanthocephalans were identified in the genus *Filisoma* Van Cleave, 1928 because of their long and slender unarmed trunk and proboscis with many hooks decreasing in size anteriorly and posteriorly, long lemnisci, double-walled proboscis receptacle with cephalic ganglion at its base, and four long tubular cement glands. A comparison with known species of *Filisoma* determined that it is new. This makes *F. caudata* n. sp. the 13th valid species of the genus and the first to be described in South America. Species of *Filisoma* appears to be present in fishes inhabiting the tropical and semitropical waters of the Indo-pacific region. The hosts, *K. incisor* and *K. sectatrix,* are nektonic, forming schools in shallow waters associated with coral reefs, sand or rocky bottom. In Western Atlantic *K. incisor* occurs from the United States to Argentina while *K. sectatrix* occurs from Canada to Santa Catarina (Brasil). These species may also occur in Eastern Atlantic coasts of Spain and Africa. They are diurnal, feeding mainly on plankton, benthonic algae, detritus, small mollusks and crustaceans (Froese and Pauly [11]).

### Description

**Fig. 1:**
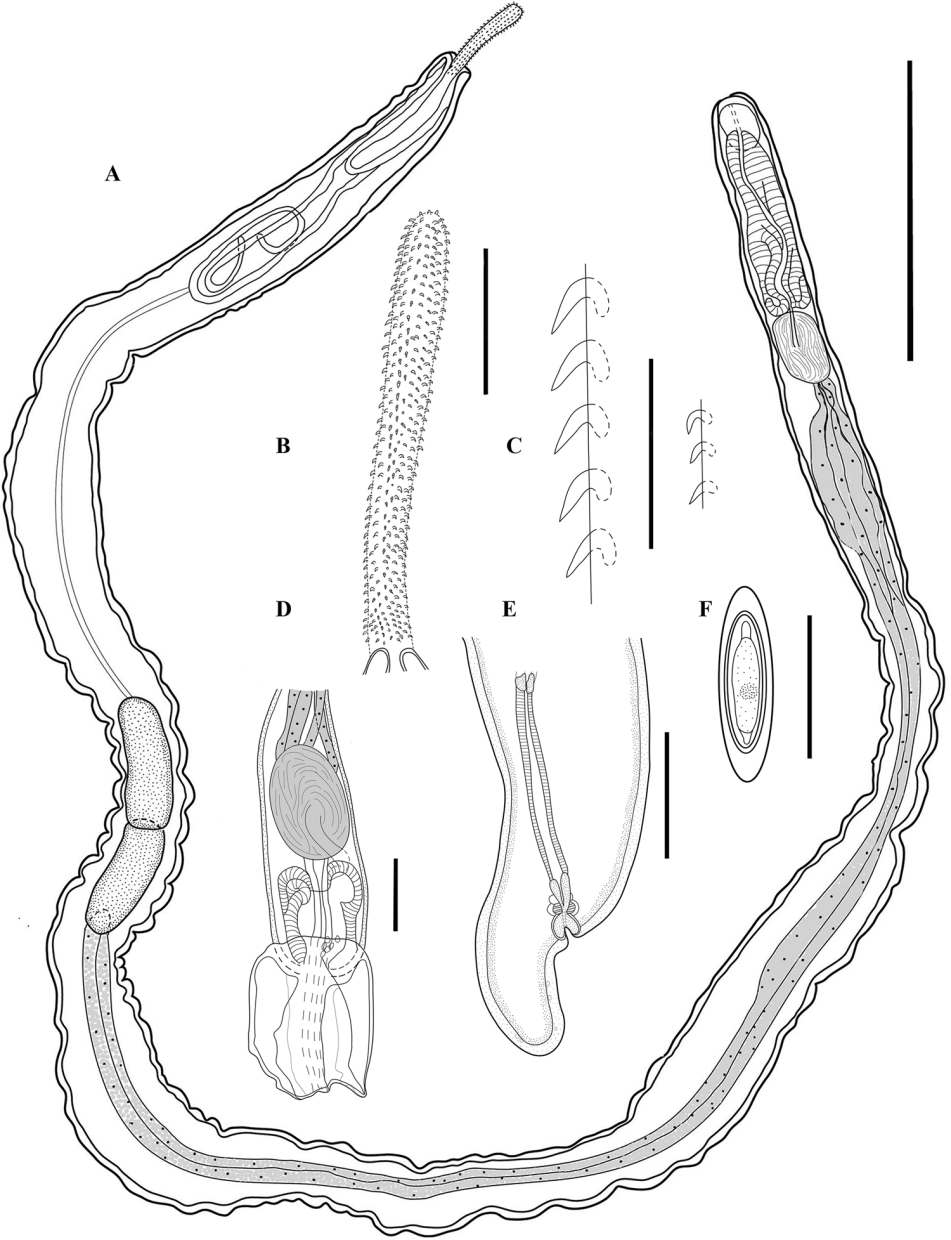
*Filisoma caudata* n. sp. light microscopy drawing. **A**: Whole body of male. **B**: Proboscis. **C**: Hook at middle of proboscis. **D**: Male posterior end. **E**: Uterus, vagina and genital pore of female. **F**: Egg. **A**: 3 mm; **B**‒**C**: 100 µm; **D**‒**E**: 500 µm; **F**: 50 µm

**Fig. 2:**
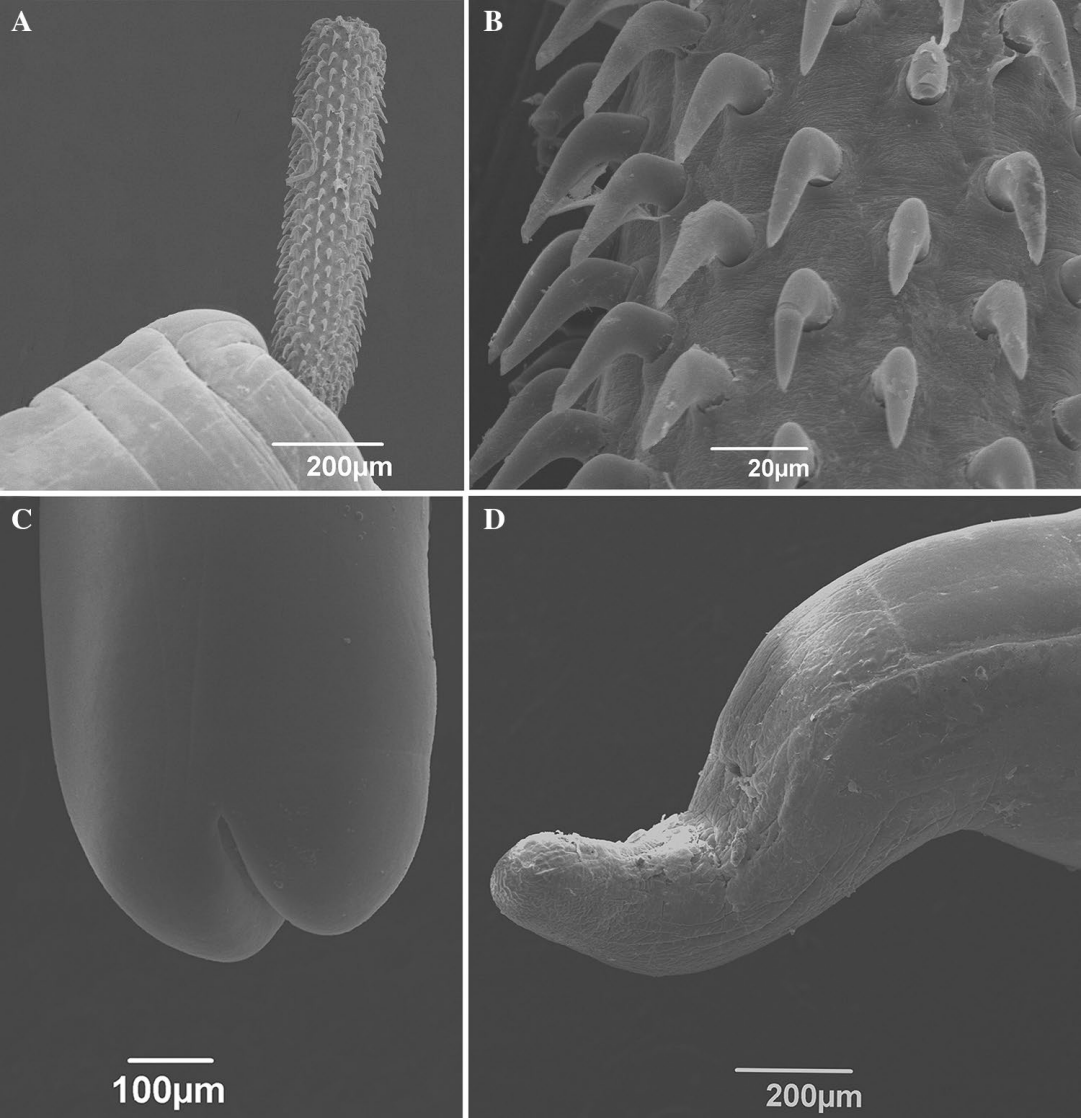
*Filisoma caudata* n. sp. scanning electron microscopy micrographs. **A** Anterior end showing proboscis with hooks. **B** Hooks in detail. **C** Male posterior end. **D** Female posterior end

*General* Cavisomidae, with characters of the genus *Filisoma*. Trunk whitish to light yellow, unarmed, long and slender (Fig. 1A). Females larger than males. Proboscis long, with 17 (16‒18) longitudinal rows each with 42 (38‒45) hooks (Figs. 1A‒C, 2A, B). Proboscis hooks directed posteriorly, with simple roots smaller than blades. Hooks uniform in shape dorsoventrally (Fig. 1C) gradually decrease in size posteriorly and measure 35 (30‒45) long anteriorly, 30 (30‒35) long at middle, and 25 (20‒30) long posteriorly (Figs. 1A‒C, 2A, B). Proboscis receptacle double walled with cerebral ganglion at its base. Lemnisci long, slender, subequal.

*Males* (based on 12 mature adults from both host species). Trunk 31 (25‒37) [26] mm × 1.15 (1.0‒1.7) [1.0] mm. Proboscis 993 (490‒1210) [970] × 134 (100‒155) [120], partially everted, with 38‒42 hooks per row. Proboscis receptacle double-walled, 1498 (1080‒1790) [1080]. Neck 154 (120‒225) [120] × 96 (70‒125) [110]. Cerebral ganglion 51 (40‒85) [40] × 84 (60‒135) [65]. Lemnisci, 5.0‒6.50 and 7.3‒7.5 mm long. Testes contiguous, pre-equatorial; anterior testis 1666 (1200‒2450) [1350] × 527 (310‒930) [450]. Posterior testis 1538 (920‒2420) [1500] × 498 (320‒760) [450] (Fig. 1A). Four tubular cement glands about half as long as trunk, 14.6 (11‒16.8) [15.8] mm long, with small oval nuclei. Safftigen’s pouch oval just anterior to copulatory bursa (Fig. 1D, invaginated in Fig. 2C).

*Females* (based on 12 gravid females from both host species). Trunk 52 (39‒65) mm × 1.3 (1.0‒1.7) mm. Proboscis 1211 (820‒1600) × 165 (125‒355) with 42 (39‒45) hooks per row. Neck 169 (125‒200) × 124 (90‒175). Proboscis receptacle 1770 (1520‒1950) long with cerebral ganglion at its base, 89 (40‒160) × 109 (60‒145). Lemnisci 4.5‒7.0 and 7.6‒8.4 mm long. Reproductive system 2.67 (2.14‒3.33) mm long with subterminal gonopore and curved posterior end (Figs. 1E, 2D). Eggs ovoid-elongate with polar prolongations of fertilization membrane, 57 (30‒70) × 19 (15‒20) (Fig. 1F).

### DNA Sequences

For the partial 18S rDNA sequences of *F. caudata* n. sp., the BLASTN results indicated 96% identity with *F. bucerium* (AF064814) considering a 70% query cover and max score 741; *Acanthocephalus lucii* (Müller, 1777) (AY830152) with 97% identity, 66% query cover and max score 741; *F. rizalinum* (JX014229) with 96% identity, 70% query cover and max score 737; *Acanthocephalus dirus* (Van Cleave, 1924) (AY830151) with 96% identity, 68% query cover and 730 max score; and *Acanthocephalus* sp. (DQ147605) with 97% identity, 66% query cover and max score 717.

For 28S rDNA gene (biggest sequence MH004455), the most similar sequences included those of *Acanthocephalus nanus* Van Cleave, 1925 (LC100043) with a 78% identity, 99% query cover and a max score of 571, *A. lucii* with 77% identity, 99% query cover and a max score of 536, *Pseudoacanthocephalus nguyenthileae* Amin, Ha & Heckmann, 2008 (KC491890), and *Pseudoacanthocephalus buffonis* (Shipley, 1903) (KC491878), both with a 79% identity, 88% query cover and a max score of 527 and 524, respectively.

For *cox*-*1* (accession number MH004408), the BLASTN results indicated for *A. dirus* (DQ089718) a 70% identity, 84% query cover and a max score of 230, *Echinorhynchida* sp. Southwell & Macfie, 1925 (EU732663) 68% identity, 95% query cover and a max score of 224 and for both *Bolbosoma caenoforme* Heitz, 1920 (KF156891) and *Bolbosoma* sp. Porta, 1908 (JX442190), 68% identity, 94% query cover and a max score of 212.

### Phylogenetic Analysis

**Fig. 3:**
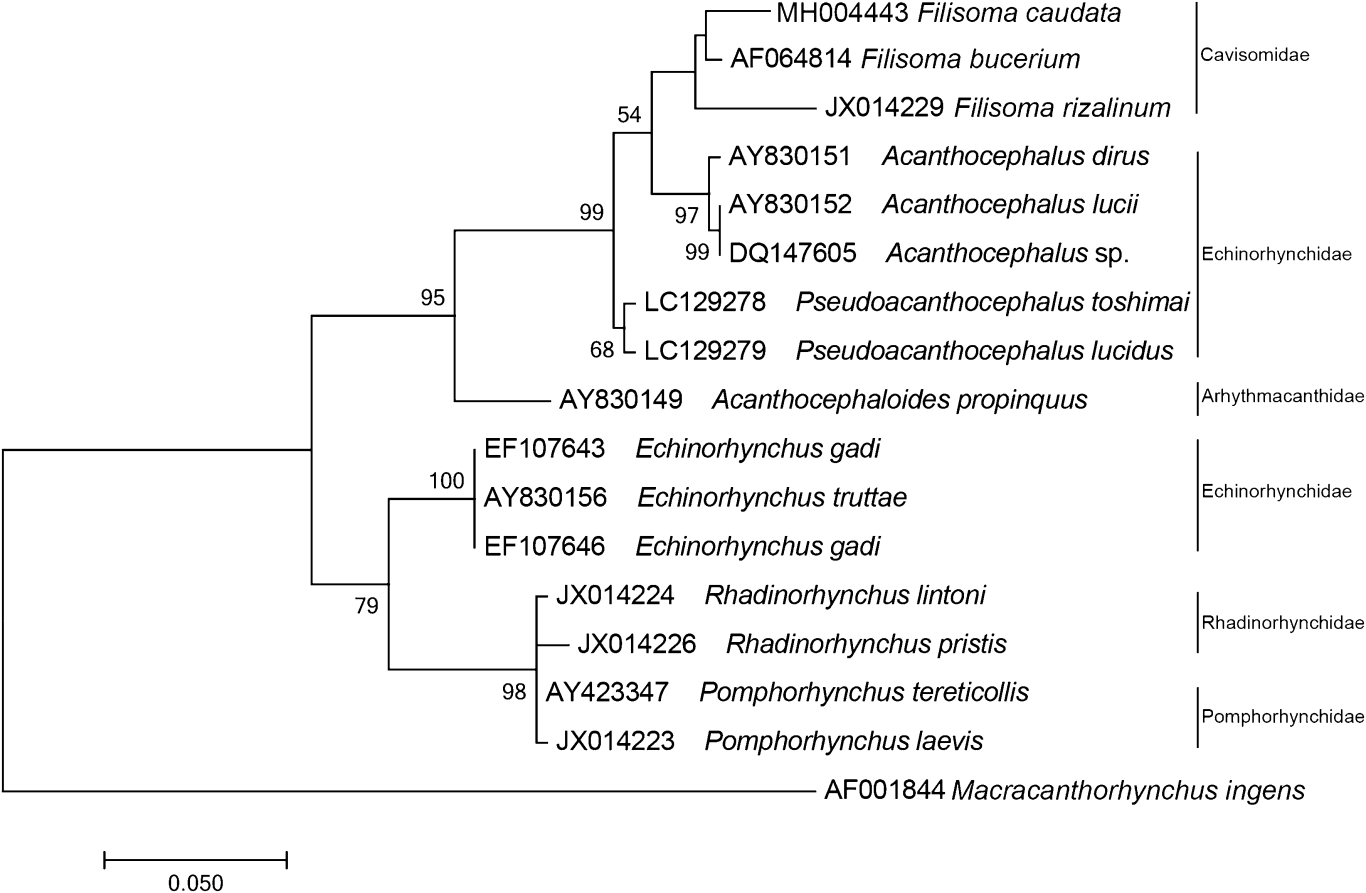
Phylogenetic reconstruction based on the Maximum likelihood analysis using 18S rDNA sequences of *Filisoma caudata* n. sp. of this work and sequences of Acanthocephala deposited in the GenBank. The numbers indicate values of bootstrap > 50%. *Macracanthorhyncus ingens* is used as an outgroup

The phylogenetic reconstruction based on the partial sequence spanning the 18S rDNA shows that our consensus sequence of *F. caudata* n. sp. is grouped with *F. bucerium* (AF064814) forming with *F. rizalinum* (JX14229) a clade of the family Cavisomidae. The sister clade with 54% support is formed by species of *Acanthocephalus.* A major clade with 99% of support encloses the two former clades and *Pseudacanthocephalus* Petrochenko, 1958 species (Fig. 3). The paraphyletic Echinorhynchidae Cobbold, 1879 family is separated between the two major clades, with species of *Echinorhynchus* Zoega in Müller, 1776 clustering with species of Rhadinorhynchidae Travassos, 1923 and Pomphorhynchidae Yamaguti, 1939 with 79% support, while the other genera grouped with Cavisomidae (Fig. 3).

**Fig. 4:**
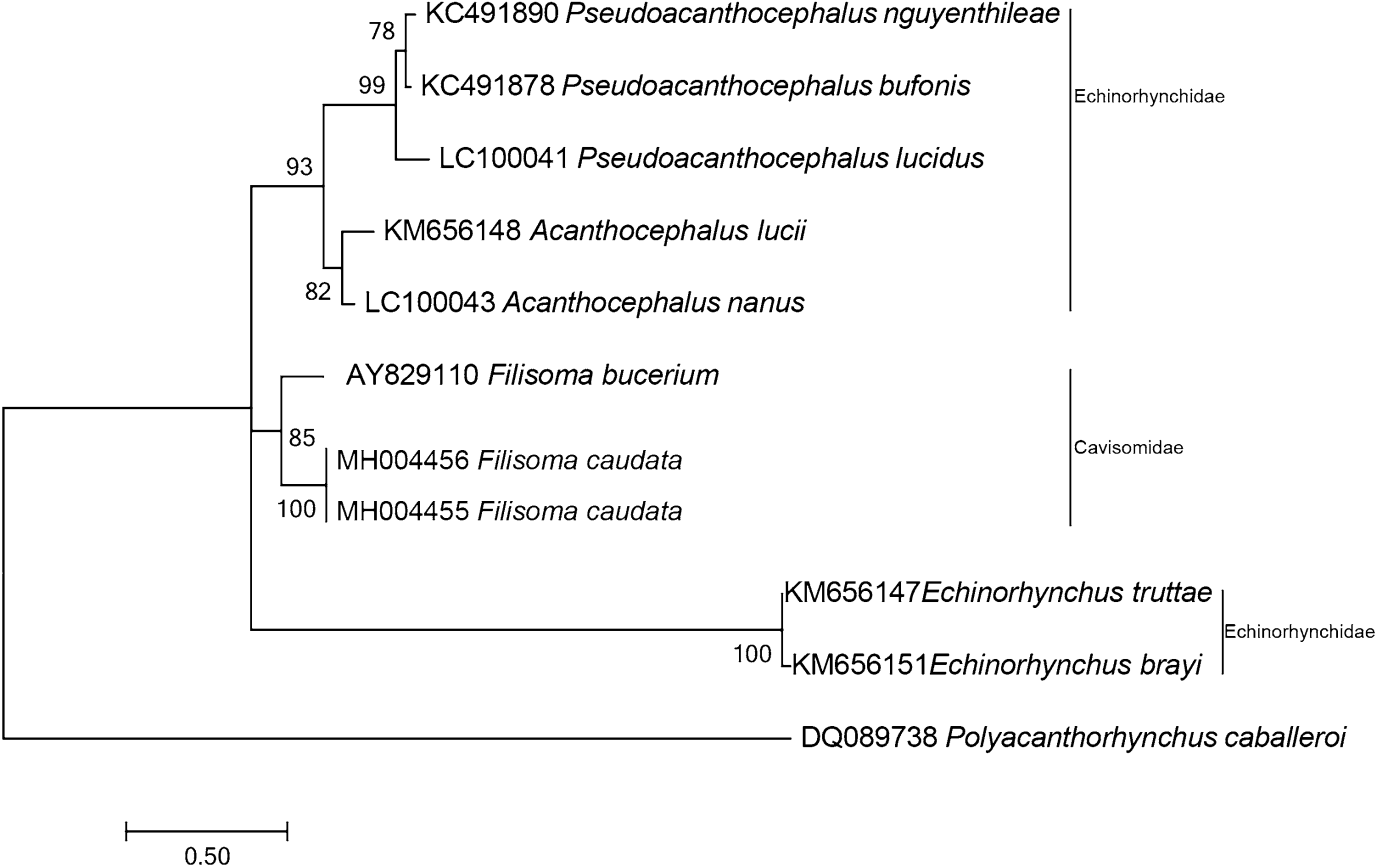
Phylogenetic reconstruction based on the Maximum likelihood analysis using 28S rDNA sequences of *Filisoma caudata* n. sp. of this work and sequences of Acanthocephala deposited in the GenBank. The numbers indicate that values of bootstrap > 50%. *Polyacanthorhynchus caballeroi* are used as an outgroup

On the partial 28S rDNA ML tree, our sequences of *F. caudata* n. sp. are grouped with *F. bucerium* (AY829110) with a statistical support of 85% in a clade of the family Cavisomidae, separated from two Echinorhynchidae clades, one formed by the species of *Acanthocephalus* and *Pseudoacanthocephalus* and the other with species of *Echinorhynchus* (Fig. 4).

**Fig. 5:**
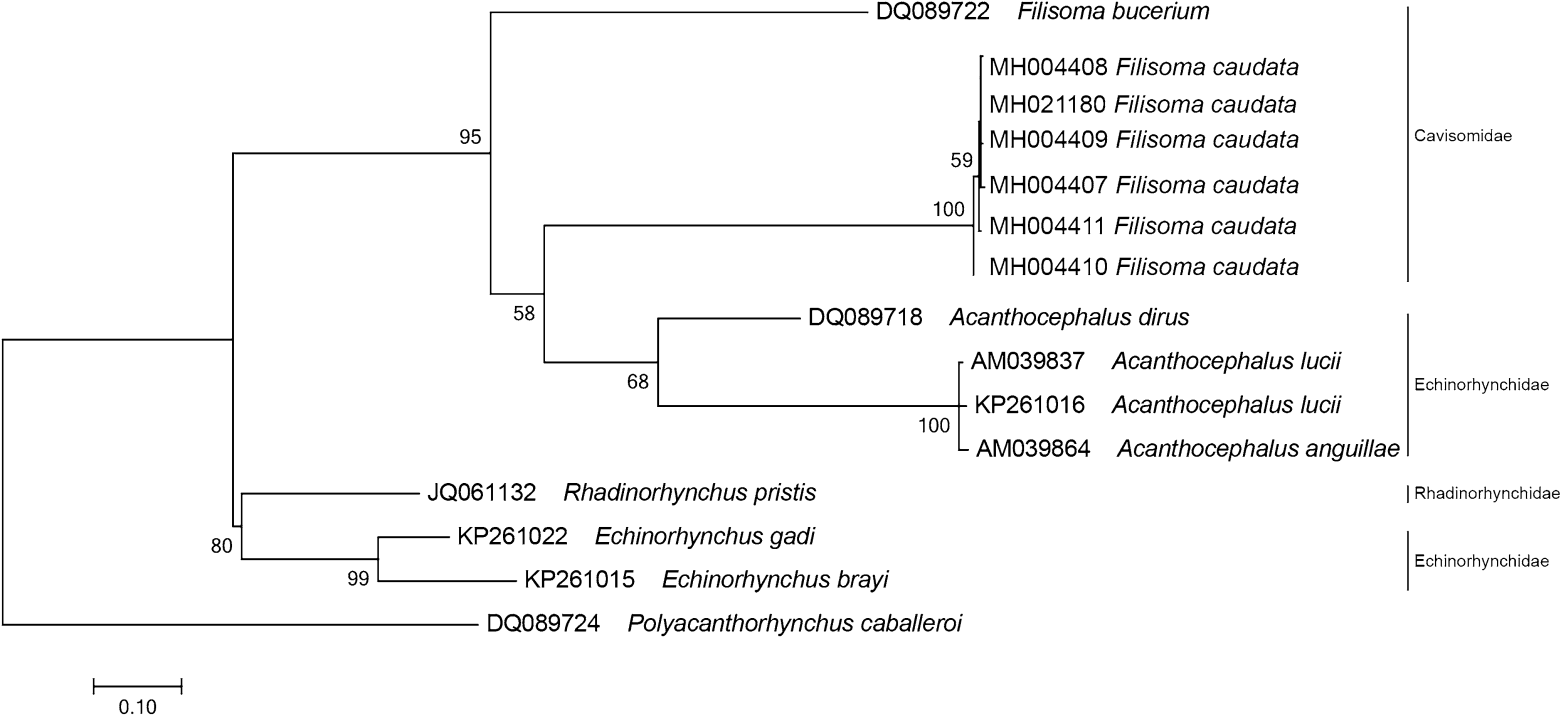
Phylogenetic reconstruction based on the Maximum likelihood analysis using partial region of the mtDNA-*cox-1* sequences of *Filisoma caudata* n. sp. of this work and sequences of Acanthocephala deposited in the GenBank. The numbers indicate that values of bootstrap > 50%. *Polyacanthorhynchus caballeroi* are used as an outgroup

On the ML reconstruction for the partial region of the mtDNA-*cox1*, all sequences of *F. caudata* clustered together with 100% of bootstrap support. The species of the genus *Acanthocephalus* appear as a sister group of our *F*. *caudata*. *F. bucerium* (DQ089722) is placed outside this cluster and shares a common ancestor with the cluster *F*. *caudata* + *Acanthocephalus* spp. Genus *Echinorhynchus* and *Rhadinorhynchus* Lühe, 1911 are placed in another cluster as sister groups with 80% of bootstrap support (Fig. 5).

### Remarks

*Filisoma caudata* n. sp. is differentiated from the closest species *F. filiformis* by the number of hooks (38‒45 hooks vs 42‒48), proboscis receptacle of males (1080‒1790 vs 2499‒3853), and a female genital pore (subterminal with a curved posterior end measuring 500‒1000 long vs terminal pore), apart from hosts and geographical distribution (Atlantic vs. Pacific Ocean). A key to the 13 species of *Filisoma* Van Cleave, 1928 is provided below.

### Key to the Species of *Filisoma* Parasites of Fish of the World

1. Proboscis with 20–37 hooks per longitudinal row…2
  – Proboscis with 38 or more hooks per longitudinal row…10
2. Proboscis with 17 or more longitudinal rows…3
  – Proboscis with 12–16 longitudinal rows…5
3. Proboscis with 17–18 longitudinal rows, with hooks uniform in shape and distribution…4
  – Proboscis with 17–19 longitudinal rows, 2 ventral rows with hooks more robust than hooks on dorsal rows, 25–26 hooks per row, lemnisci shorter than receptacle and long cement glands (3.56–16.02 mm), in *Scatophagus argus* (L. 1766) in Fiji Islands…F*ilisoma longcementglandatus*
4. Proboscis with 18 longitudinal rows and 20 hooks per row, receptacle 1.84–2.4 mm long, lemnisci almost the same length of receptacle, in *Acanthocybium solandri* (Curvier, 1832) from China…*Filisoma acanthocybii*
  – Proboscis with 17–18 rows and 23–24 hooks per row, receptacle up to 1.01 mm long, lemnisci longer than proboscis receptacle and short cement glands (up to 2 mm), in *Saurus myops* (Forster, 1801) from India…*Filisoma inglisi*
5. Dorsal row of hooks different in size…6
  – Dorsal row of hooks uniform in size…7
6. Proboscis 0.8–0.95 mm long with 16 longitudinal rows of 23–24 hooks each, two submedian dorsal rows of hooks larger, trunk 23–27.5 mm, in *S. argus* from Philippines…*Filisoma rizalinum*
  – Proboscis 1.1 mm long with 14–16 longitudinal rows of 26–32 hooks each, dorsal hooks thicker and less pointed than ventral hooks, trunk 115 mm, in *S*. *argus* from India…*Filisoma scatophagusi*
7. Proboscis with 12–14 longitudinal rows…8
  – Proboscis with 16 longitudinal rows, 28 hooks per row uniform in shape and leminisci shorter than proboscis receptacle, in *Microcanthus strigatus* (Curvier, 1831) from Taiwan…*Filisoma microcanthi*
8. Without two ventral protuberances at vulva…9
  – With two ventral protuberances at vulva, 24–28 hooks per row, body 20–30 mm, receptacle 1.3 mm long and leminisci the same length of proboscis receptacle, in *S*. *argus* from India…*Filisoma indicum*
9. Trunk 7.5 mm, proboscis 1.76 mm long with 14 longitudinal rows each with 28 hooks per row, parasitizes *Oplegnathus fasciatus* (Temminck & Schlegel, 1844) from China…*Filisoma oplegnathi*
  – Trunk 8–8.4 mm, proboscis 1.14 long with 14 longitudinal rows each with 22 hooks per row; in *Atropus atropos* (Bloch & Schneider, 1801) from China…*Filisoma atropi*
10. Proboscis without 4 to 5 basal hooks configured in spiral…11
  – Proboscis with 4 to 5 basal hooks configured in spiral, 17–20 rows, 38 hooks per row, body 76–95 mm, receptacle up to 2.8 mm and leminisci shorter than proboscis receptacle, parasitize *Kyphosus sectatrix* from USA…*Filisoma fidum*
11. Median dorsal longitudinal row of proboscis uniform…12
  – Median dorsal longitudinal row of proboscis with blunt, hornlike hooks, proboscis 1.5–2.0 mm long with 16 longitudinal rows, 38–48 hooks per longitudinal row, body 45–60 mm, receptacle up to 4.2 mm and leminisci with the same length of proboscis receptacle, parasitize *Kyphosus elegans* (Peters, 1869) from Mexico…*Filisoma bucerium*
12. Proboscis 1.01–1.54 mm long with 16–18 longitudinal rows, 42–48 hooks per row, body 28–70 mm, receptacle with 2.49–3.63 mm, leminisci longer than proboscis receptacle and female genital pore terminal, parasitize *Kyphosus bigibbus* Lacepède, 1801 and *Kyphosus vaigiensis* (Quoy & Gaimard, 1825) from Australia…*Filisoma filiformis*
  – Proboscis 0.49–1.21 mm long with 16–18 longitudinal rows, 38–45 hooks per row, body 25–65 mm, receptacle with 1.08–1.95, leminisci longer than proboscis receptacle and female genital pore subterminal with posterior body end curved 500–1,000 long tail, parasitize *Kyphosus incisor* and *Kyphosus sectatrix* from Brasil…*Filisoma caudata* n. sp.

## Discussion

Four species of *Filisoma* parasitize kyphosid fish each of which has more than 28 proboscis hooks per longitudinal row: (1) *F. filiformis* from *Kyphosus bigibbus*, *Kyphosus sydneyanus* Günther, 1886 and *Kyphosus vaigiensis* off Australia, (2) *F. bucerium* from *Kyphosus elegans* off the Pacific coast of México, (3) *F. fidum* Van Cleave & Manter, 1947 from *K. sectatrix* off the Atlantic coast of Florida, United States [1, 2, 5, 6] and (4) *F*. *microcanthi* from *Microcanthus strigatus* from Taiwan [22]. *F. caudata* n. sp. differs from *F. filiformis* by the subventral female gonopore followed by long posterior end (tail) and by the smaller difference in the number of hooks, as well as host and geographical distribution. *F. bucerium* can be primarily differentiated from the new species by having heavy and blunt modified dorsal hooks at middle of proboscis, lemnisci about the same length of receptacle and different shapes of the female tail [1, 6]. *F. fidum,* although sharing the same host species with *F. caudata* n. sp., occurs far apart in the north Atlantic ocean (Florida) and can be differentiated by their larger size of males (76 × 25‒37 mm) and females (95 × 39‒65 mm), longer proboscis receptacle (up to 2.8 mm) and longer testis (3.5 × 1.6 mm). The absence of a long female tail can also be an inferred difference, considering that this character was not mentioned in the original description. The above key further differentiates our new species from these four species and others of the genus from other host species.

The phylogenetic analysis of the 28S rDNA sequences (Fig. 4) grouped *F. caudata* n. sp. with *F. bucerium* (AY829110) forming a clade of the family Cavisomidae between two clades in the family Echinorhynchidae confirming that different genera of Echinorhynchidae are paraphyletic [23–25]. Our phylogenetic tree for 28S rDNA is similar to those of Braincovich et al. [26] and Gárcia-Varela and Nadler [23], where the family Cavisomidae grouped close to the Echinorhynchidae genus *Acanthocephalus*. Although García-Varela and Nadler [23] commented that the 28S rDNA is not the most appropriate for taxonomic studies at the generic level, the 18S rDNA sequences appear to be more suitable to infer phylogenies among Acanthocephalans. The clade with the Cavisomidae had a good statistical support for the genus *Filisoma*. The phylogenetic analysis of the 18S rDNA sequences (Fig. 3) confirmed a clade of Cavisomidae with *F. caudata* n. sp. and *F. bucerium* (AF064814) that are well separated from Echinorhynchidae, Arhythmacanthidae (see Braincovich [26]), Rhadinorhynchidae, and Pomphorhynchidae.

Our phylogenetic analysis of the mtDNA *cox*-*1* gene (Fig. 5) also grouped *Filisoma* spp. as sister groups of *Acanthocephalus* spp. However, more sequences of other species of Cavisomidae are necessary to better understand the relationship between species of these two genera, since *Filisoma* is the only genus with sequences available in the GenBank. Benesh et al. [27] discussed the reliability of the use of mitochondrial DNA amplified with universal primers for taxonomy since amplification could result in fragments of nuclear pseudogenes that have sequences similar to mitochondrial genes. In this work, the results of the phylogeny did not differ much in topology from the analysis made with nuclear genes indicating that the mitochondrial sequences used are reliable to infer phylogenies. The greater impediment to robust analysis was the reduced number of sequences deposited for the family Cavisomidae in the GenBank.

Amin [3] discussed the need of reevaluation of the families of Palaeacantocephala considering that the classification of families based only on morphology, e.g., the number of cement glands, can be doubtful. After Braincovich et al. [26] families with these characteristics may not be related as Cavisomidae and Rhadinorhynchidae, both usually with four cement glands that are grouped into different clades. Gymnorhadinorhynchidae Braicovich et al., 2014, for example, with four cement glands, group with Transvenidae Pichelin & Cribb, 2001, which has only two cement glands [26].

The molecular data analyzed also suggest that families of Palaeacanthocephala must be reevaluated, since the delineation of monophyletic families was not clear in any of the topologies obtained, especially for species of Echinorhynchidae. The lack of sequences of different genera of Acanthocephala demonstrates that the use of molecular tools in defining species of Acanthocephala is still scarce and a large number of studies still describe and redescribe species based only on morphology [2, 4, 28–32]. For Cavisomidae, for example, there are genetic sequences of only three species available in the GenBank, all of them from the genus *Filisoma* (*F. bucerium*, *F. rizalinum* and now *F. caudata* n. sp.). Therefore, new integrative studies with morphological, molecular and geographical distribution data help to determine species with reliability and are necessary to better understand the classification of acanthocephalans [23, 25]. Keys to species of *Filisoma* were previously provided by Van Cleave and Manter [1], Amin and Nahhas [5] and Weaver and Smales [2]. An updated key for species of *Filisoma* including *F. caudata* n. sp. is now provided.
